# Microbial Community Functional Structures in Wastewater Treatment Plants as Characterized by GeoChip

**DOI:** 10.1371/journal.pone.0093422

**Published:** 2014-03-26

**Authors:** Xiaohui Wang, Yu Xia, Xianghua Wen, Yunfeng Yang, Jizhong Zhou

**Affiliations:** 1 Environmental Simulation and Pollution Control State Key Joint Laboratory, School of Environment, Tsinghua University, Beijing, China; 2 Department of Environmental Science and Engineering, Beijing University of Chemical Technology, Beijing, China; 3 Institute for Environmental Genomics and Department of Microbiology and Plant Biology, University of Oklahoma, Norman, Oklahoma, United States of America; 4 Earth Sciences Division, Lawrence Berkeley National Laboratory, Berkeley, California, United States of America; National University of Singapore, Singapore

## Abstract

**Background:**

Biological WWTPs must be functionally stable to continuously and steadily remove contaminants which rely upon the activity of complex microbial communities. However, knowledge is still lacking in regard to microbial community functional structures and their linkages to environmental variables.

**Aims:**

To investigate microbial community functional structures of activated sludge in wastewater treatment plants (WWTPs) and to understand the effects of environmental factors on their structure.

**Methods:**

12 activated sludge samples were collected from four WWTPs in Beijing. A comprehensive functional gene array named GeoChip 4.2 was used to determine the microbial functional genes involved in a variety of biogeochemical processes such as carbon, nitrogen, phosphorous and sulfur cycles, metal resistance, antibiotic resistance and organic contaminant degradation.

**Results:**

High similarities of the microbial community functional structures were found among activated sludge samples from the four WWTPs, as shown by both diversity indices and the overlapped genes. For individual gene category, such as *egl, amyA*, *lip*, *nirS*, *nirK*, *nosZ*, *ureC*, *ppx*, *ppk*, *aprA*, *dsrA*, *sox* and *benAB,* there were a number of microorganisms shared by all 12 samples. Canonical correspondence analysis (CCA) showed that the microbial functional patterns were highly correlated with water temperature, dissolved oxygen (DO), ammonia concentrations and loading rate of chemical oxygen demand (COD). Based on the variance partitioning analyses (VPA), a total of 53% of microbial community variation from GeoChip data can be explained by wastewater characteristics (25%) and operational parameters (23%), respectively.

**Conclusions:**

This study provided an overall picture of microbial community functional structures of activated sludge in WWTPs and discerned the linkages between microbial communities and environmental variables in WWTPs.

## Introduction

Biological activated sludge process is the most widely used biological process for treating municipal and industrial wastewater. By enriching selected functional microorganisms in the activated sludge of wastewater treatment plant (WWTP), microbial activities in the sludge community are accelerated, enabling removal of oxygen-depleting organics, toxics, and nutrients. Biological WWTPs must be functionally stable to continuously and steadily remove contaminants which rely upon the activity of complex microbial communities [1], [2]. Therefore, a better understanding of the microbial community structure and functional genes of activated sludge in WWTPs can help elucidate the mechanisms of biological pollutant removal and improve the treatment performance and operational stability [3], [4].

Although microbial communities of activated sludge in WWTPs have been intensively studied [5]–[11], most of such efforts have been focused only on microbial phylogenetic composition. Knowledge is still lacking in regard to microbial community functional structures and their linkages to environmental variables. To date, there have been only a limited number of studies that characterized the overall functional profiles and metabolic pathways in the activated sludge of WWTPs using high-throughput sequencing [12], [13].

Functional gene arrays, which contain genes encoding key enzymes involved in a variety of biogeochemical cycling processes [14], can be used to study the overall microbial functional potentials of activated sludge in WWTPs. GeoChip 4.2, a powerful functional gene array, contains 83,992 oligonucleotide (50-mer) probes targeting 152,414 genes in 410 gene categories involved in nitrogen, carbon, sulfur and phosphorus cycling, metal resistance, antibiotic resistance as well as organic contaminant degradation [15], [16]. GeoChip has been widely used to examine microbial community functional structures in various environmental samples, such as soil and sediments [17]–[19], groundwater [20], acid mine drainage[21], deep-sea water [15], ocean crust [15], and hydrothermal vent [22].

In this study, 12 activated sludge samples from four WWTPs in Beijing were analyzed by GeoChip 4.2 to address the following two questions: (i) What were the microbial community functional structures of activated sludge in WWTPs? (ii) How do environmental factors and operational parameters affect microbial community functional structures? Our results revealed high similarities of microbial functional communities among activated sludge samples from the four WWTPs, and microbial functional potentials were highly correlated with water temperature, dissolved oxygen (DO), ammonia concentrations and loading rate of chemical oxygen demand (COD).

## Materials and Methods

### WWTPs and Sampling

Anaerobic/anoxic/aerobic (A^2^O) is the most commonly used process for wastewater treatment in China, thus we examined four A^2^O WWTPs in Beijing. Activated sludge samples were taken from the aeration tank of each WWTP once a day for three consecutive days in the summer of 2011. Therefore, three replicate samples were available from each WWTP, resulting in a total of 12 activated sludge samples. No specific permits were required for the described field studies. We confirm that: i) the locations were not privately-owned or protected in any way; and ii) the field studies did not involve endangered or protected species.

For microbial community analysis, each sample was dispensed into a sterile Eppendorf tube and centrifuged at 14,000 *g* for 10 min. After decanting the supernatant, the pellet was stored at −80°C prior to further analysis. Meanwhile, various chemical parameters such as COD, total nitrogen (TN), ammonia, total phosphorus (TP), pH and conductivity were analyzed according to standard methods [23]. Concentrations of chromium, cobalt, nickel, copper, zinc, and cadmium were measured by inductively coupled plasma mass spectrometry (ICP-MS)(Thermo Fisher Scientific, Waltham, MA, USA).

### DNA Purification and GeoChip Hybridization

Microbial genomic DNA was extracted from the activated sludge samples by combining freeze-thawing and sodium dodecyl sulfate (SDS) for cell lysis as previously described [24]. Crude DNA was purified using the Wizard SV Genomic DNA Purification Kit (Promega, Madison WI, USA). The quality of purified DNA was assessed based on the ratios of 260/280 nm and 260/230 nm absorption measured by an ND-1000 spectrophotometer (Nanodrop Inc., Wilmington, DE, USA) and agarose gel electrophoresis. The quantity of community DNA was determined with Quant-It PicoGreen kit (Invitrogen, Carlsbad, CA, USA).

Purified DNA (1 μg) was labeled with Cy5, purified and resuspended in 10 μl hybridization solution as previously described (Lu *et al*. 2012). Then it was hybridized with GeoChip 4.2 on a MAUI hybridization station (BioMicro, Salt Lake City, UT, USA) at 42°C with 40% formamide for 16 hours. After hybridization, microarrays were scanned (NimbleGen MS200, Madison, WI, USA) at a laser power of 100%. The ImaGene version 6.0 (Biodiscovery, El Segundo, CA) was then used to determine the intensity of each spot, and identify poor-quality spots. The GeoChip raw data has been uploaded to the NCBI GEO with the accession number GSE54055.

### Data Analysis

Raw data from ImaGene were submitted to Microarray Data Manager (http://ieg.ou.edu/microarray/) and analyzed using the data analysis pipeline with the following major steps: (i) The spots flagged as 1 or 3 by Imagene and with a signal to noise ratio (SNR) less than 2.0 were removed as low quality spots; (ii) After removing the bad spots, normalized intensity of each spot was calculated by dividing the signal intensity of each spot by the mean intensity of the microarray; (iii) If any of replicates had more than two times the standard deviation, this replicate was moved as an outlier [18].

Hierarchical cluster analysis was performed using the unweighted pairwise average-linkage clustering algorithm in the CLUSTER software and visualized in TREEVIEW software [25]. Canonical correspondence analysis (CCA) was used to examine the relationships of microbial communities and environmental variables. The significant environmental variables were identified by a forward selection procedure using Monte Carlo permutations ((999 permutations with a *p*-value<0.05). In addition, Variance inflation factors (VIFs; a measure for cross-correlation of explanatory variables) were checked and eliminated if VIFs were more than 20 [26].Based on partial CCA, variance partitioning analysis (VPA) was performed to attribute the variation observed in the microbial communities to the environmental variables. CCA and VPA were performed by the vegan package in R 2.14.0 (R Development Core Team, 2011).

## Results

### Performance of WWTPs

Details of the influent and effluent characteristics and operational parameters of the four WWTPs are listed in Table S1 and S2 in File S1. Chemical oxygen demand (COD) in the influents ranged from 257 to 452 mg/L, while total nitrogen (TN) concentrations were between 46 and 64 mg/L. The COD removal efficiencies were more than 90% in all systems, and TN removal efficiencies varied from 68% to 75%. The removal efficiencies of COD and TN indicated that the four WWTPs were functionally stable.

### Overall Microbial Community Functional Structure

**Figure 1:**
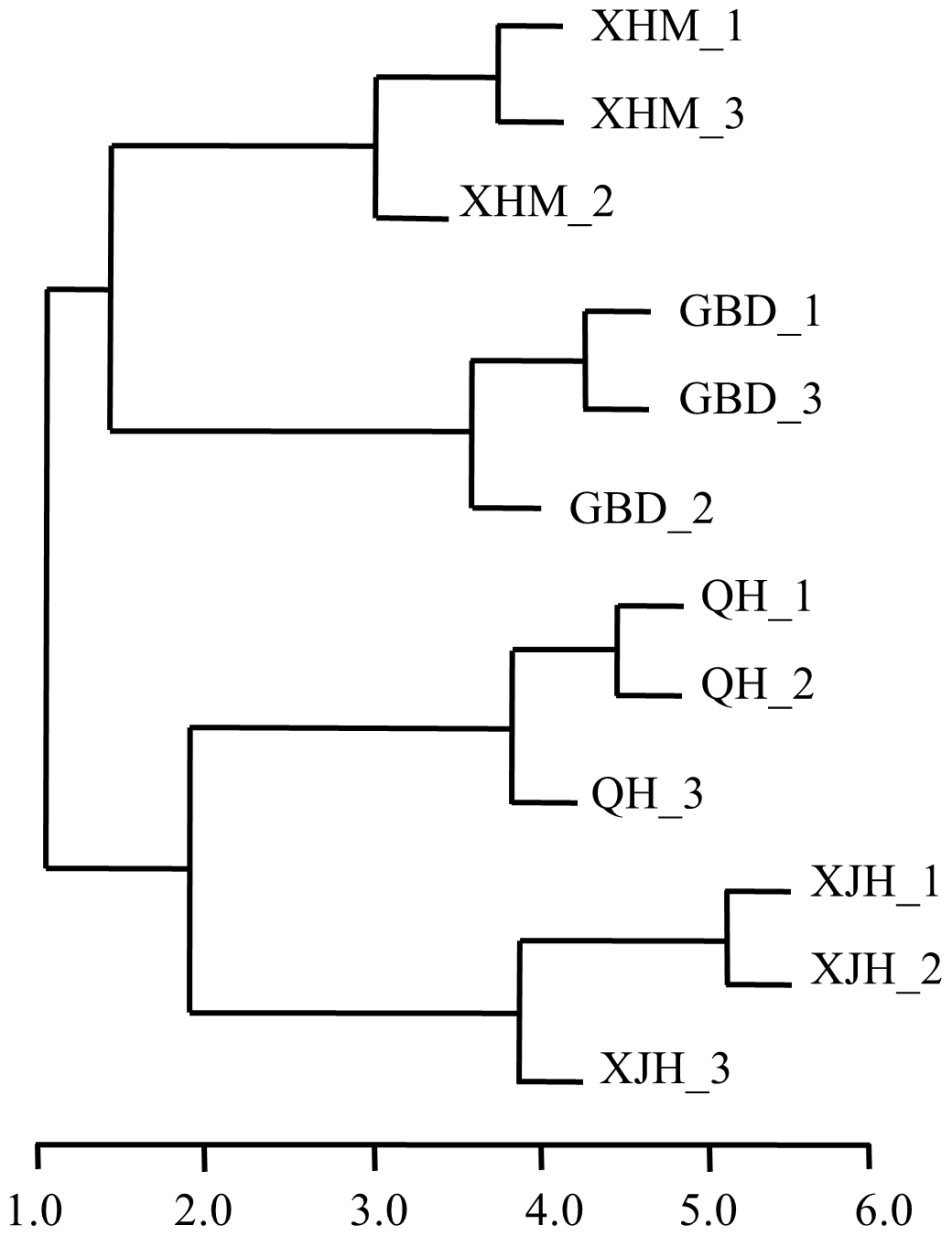
Hierarchical cluster analysis of functional genes in 12 activated sludge samples from four WWTPs named GBD, QH, XHM and XJH. Each sample is named after WWTP plant with “_1”, “_2”, or “_3” that indicates one of three replicate samples from each plant.

Microbial community functional structures of 12 activated sludge samples from four WWTPs were analyzed with GeoChip 4.2 A total of 29,124 functional genes were detected. For individual samples, the numbers of detected genes were between 18,847 and 24,268 (Table 1). Hierarchical cluster analysis showed that the three replicate samples from each WWTP were clustered together, and the distances among the three replicate samples was always <2 (Figure 1). The samples from different WWTPs were well separated, and the distances among the four WWTPs were generally>2.

**Table 1:** Percentage of overlapping genes and diversity indices of all functional genes in activated sludge samples.

sample	GBD_1	GBD_2	GBD_3	QH_1	QH_2	QH_3	XHM_1	XHM_2	XHM_3	XJH_1	XJH_2	XJH_3
GBD_1												
GBD_2	84.67%											
GBD_3	89.16%	86.65%										
QH_1	69.41%	71.45%	71.96%									
QH_2	67.39%	69.49%	69.22%	85.78%								
QH_3	65.12%	68.03%	67.38%	81.68%	80.53%							
XHM_1	79.10%	80.08%	81.10%	72.82%	69.85%	67.72%						
XHM_2	75.56%	78.38%	77.87%	71.42%	68.90%	68.33%	85.82%					
XHM_3	77.18%	78.64%	79.68%	74.95%	72.89%	71.38%	88.14%	82.43%				
XJH_1	73.92%	77.03%	74.81%	71.96%	71.38%	71.08%	75.72%	76.30%	76.37%			
XJH_2	74.11%	76.22%	74.91%	72.62%	72.29%	72.01%	74.94%	75.19%	76.71%	89.15%		
XJH_3	67.12%	69.41%	67.89%	70.21%	71.17%	70.94%	67.74%	69.71%	69.96%	79.99%	80.27%	
Richness^a^	23064	22434	23554	20862	20610	19688	24268	22831	23413	20774	20838	18847
H^b^	10.1	10.0	10.1	10.0	9.9	9.9	10.1	10.0	10.1	10.0	10.0	9.9
1/D^c^	23301.2	22682.7	23805.7	21049.3	20777.9	19855.9	24452.9	23022.1	23624.7	20959.6	21033.5	18984.4
Evenness^d^	0.996	0.996	0.996	0.996	0.995	0.995	0.994	0.995	0.995	0.995	0.995	0.995

a Detected gene number.

b Shannon–Weiner index, higher number represents higher diversity.

c Reciprocal of Simpson's index, higher number represents higher diversity.

d Evenness index.

To assess α-diversity of microbial communities, Shannon-Weaver index (H), Simpson's (1/D) and evenness were calculated (Table 1). The values of H were very similar across the 12 samples, ranging from 9.9 to 10.1. Consistently, the Simpson's (1/D) and evenness did not show significant differences among these four plants.

The overlapping genes were also determined. A large percentage (65–89%) of detected genes were shared among the samples (Table 1), indicative of high similarities of microbial community functional structures among activated sludge of four WWTPs. In consistency to the results of hierarchical clustering analysis, the percentages of the overlapping genes in samples from the same WWTP were higher than those from different WWTPs. For example, sample GBD_1 had 85% and 89% of the genes in common with sample GBD_2 and GBD_3 respectively, but 65% in common with QH_3.

At the phylogenetic level, 87.6–88.7% genes were derived from bacteria, 1.9–2.0% from archaea, and 8.7–9.7% from fungi. *Proteobacteria* was the predominant phylum of bacteria, constituting 50.8–52.2% of all detected microorganisms. *Actinobacteria*, *Firmicutes*, *Cyanobacteria* and *Bacteroidetes* were the subdominant bacterial groups, each containing 14.8–15.5%, 4.6–5.1%, 1.8–2.1% and 1.3–1.6% of detected microorganisms, respectively. *Ascomycota* was the most abundant phylum of fungi, constituting 6.3–7.4% of all detected microorganisms, followed by *Basidiomycota* (1.5–1.7%) as the second most abundant fungal group. In addition, *Euryarchaeota*, containing about 1.5% of detected microorganisms, was the most predominant phylum within archaea. (Table S3 in File S1). Taken together, these results indicated overall functional structures as well as phylogenetic diversities of microbial communities within WWTPs appeared to be quite high.

### Carbon Cycling Genes

**Figure 2:**
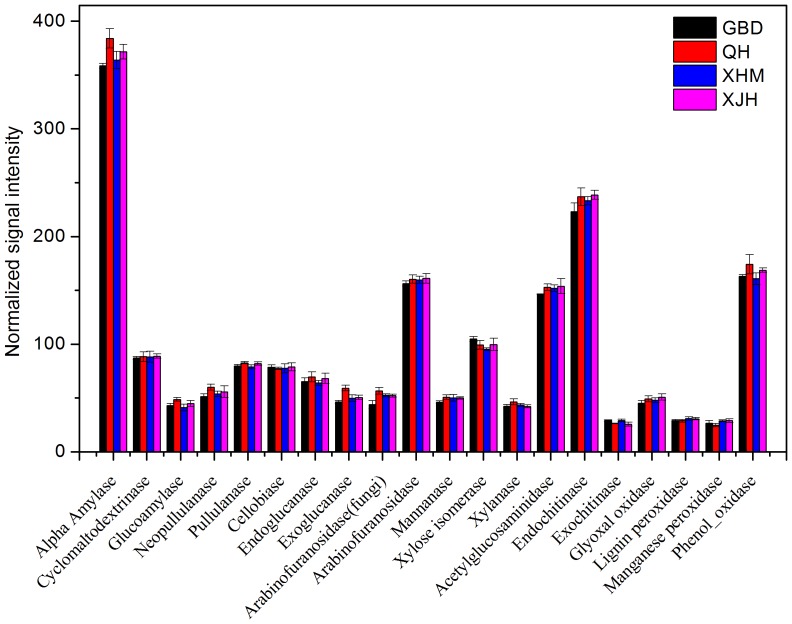
The normalized signal intensity of detected key genes involved in carbon cycling. The signal intensity for each functional gene was the average of the signal intensity from all the replicates. All data are presented as mean ± SE.

Carbon cycling, especially carbon degradation, is a crucial biochemical process in WWTPs. The rate of carbon degradation depends on a number of factors, including the availability and types of carbon substrates as well as the microbial community [27]. Among detected carbon cycling genes, 3,375 genes were involved in the degradation of complex carbon substrates such as cellulose, starch, hemicelluloses, chitin and lignin. Generally, relative abundances and numbers of all detected carbon degradation genes were similar among activated sludge of four WWTPs (Figure 2 and Table S4 in File S1).

Endoglucanase are responsible for the initial steps of cellulose degradation. A total of 41 *egl* genes encoding endoglucanase were detected, and about half of them (20 genes) were commonly shared by all samples (Figure S1 in File S1). Nearly all of the *egl* genes were derived from isolated microorganisms, including the probes from the sequenced 149121623 (protein id number) of *Methylobacterium* sp. 4–46, 197934254 of *Streptomyces sviceus* ATCC 29083, and 171059025 of *Leptothrix cholodnii* SP-6.

α-Amylase functions to hydrolyze starch to yield glucose and maltose, which plays an important role in starch degradation. A total of 366 *amyA* genes encoding α-amylase were detected, among which 156 genes were commonly shared across all samples. Almost all the detected *amyA* genes were derived from isolated organisms such as 113897923 of *Herpetosiphon aurantiacus* ATCC 23779, 50842594 of *Propionibacterium acnes* KPA171202, 240169491 of *Mycobacterium kansasii* ATCC 12478.

Xylanase is an enzyme that breaks down hemicellulose, which is a major component of plant cell walls. 33 xylanase genes were detected, and 13 of them were shared by all samples (Figure S2 in File S1). Most of xylanase genes were from isolates such as 113935746 of *Caulobacter* sp. K31, 116098389 of *Lactobacillus brevis* ATCC 367, 68230042 of *Frankia* sp. EAN1pec, etc. Also a total of 422 genes encoding chitin degradative enzymes, including endochitinase, acetylglucosaminidase and exochitinase, were found in all 12 samples. For genes encoding endochitinase, 99 out of 239 genes were shared by all samples.

A total of 268 genes related to lignin degradation were detected, including genes encoding glyoxal oxidase (*glx*), ligninase (*lip*), manganese peroxidase (*mnp*) and phenol oxidase (*lcc*). Many of these genes were detected across all samples. For example, 13 out of 28 *lip* genes were shared by all samples and all of them were derived from isolates (Figure S3 in File S1). Similarly, almost all genes of *glx*, *mnp* and *lcc* were from isolated microorganisms rather than uncultured microorganisms.

### Nitrogen Cycling Genes

**Figure 3:**
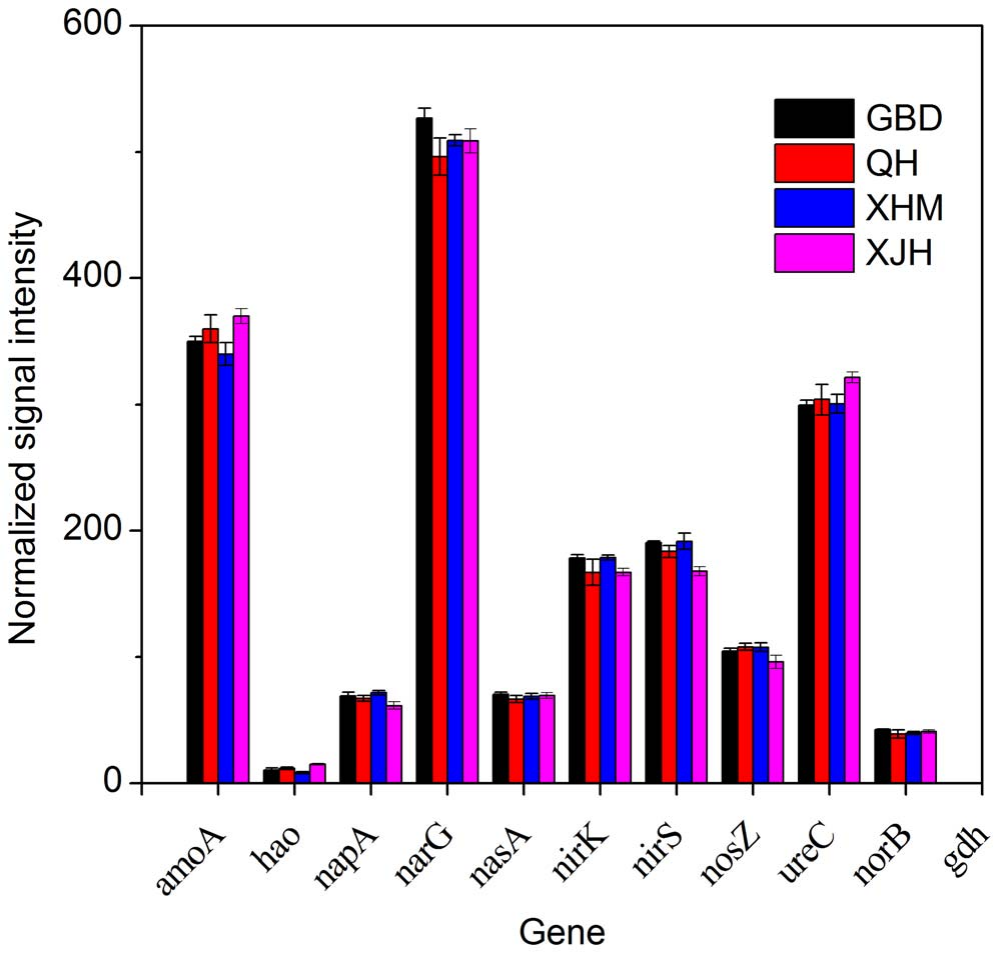
The normalized signal intensity of detected key genes involved in nitrogen cycling. The signal intensity for each functional gene was the average of the signal intensity from all the replicates. All data are presented as mean ± SE.

As nitrogen removal is one of the most important functions in WWTPs, we examined the nitrogen cycling genes and their relationship to nitrogen concentrations. A total of 2,055 genes involved in ammonification, nitrification, denitrification and assimilatory nitrate/nitrite reduction were detected by GeoChip 4.2. Similar to carbon degradation genes, the relative abundances of detected nitrogen cycling genes were similar among activated sludge of four WWTPs (Figure 3).

Of 197 detected *nirS* genes encoding nitrite reductases, 65 were present in all samples. 7 genes were derived from isolates, while the majority of genes were derived from environmental clones in various environments. The results of Mantel test showed that the abundance of *nirS* genes was positively correlated to the influent concentrations of TN (r = 0.489, *P*<0.01) and ammonia (r = 0.498, *P*<0.01) (Table S5 in File S1).

For *nirK* genes encoding nitrite reductases, 184 genes were detected. Among them, 62 genes were present in all samples. Most of them were from laboratory clones, while only 10 genes were derived from isolates. The results of Mantel test indicated that *nirK* genes were positively correlated to the concentrations of influent TN (r = 0.421, *P*<0.01) and ammonia (r = 0.358, *P*<0.05) (Table S5 in File S1).

**Figure 4:**
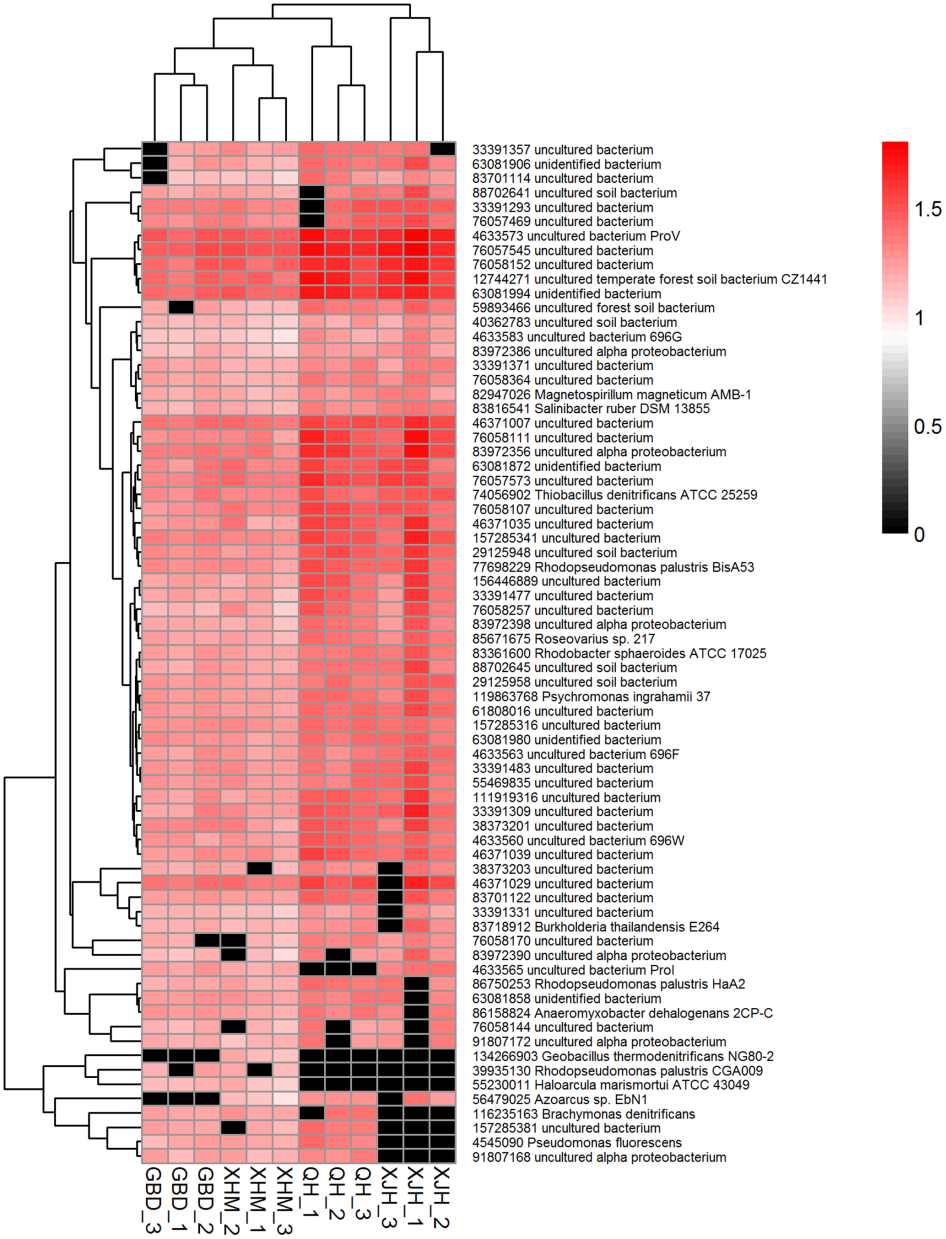
The hierarchical cluster analysis of *nosZ* genes encoding nitrous oxide reductases. The protein id number and its derived organism for each gene are indicated. The color intensity of each panel shows the normalized signal intensity of individual genes, referring to color key at the top right.

For *nosZ* genes encoding nitrous oxide reductases, 110 genes were detected. Among them, 43 were shared by all samples. 94 *nosZ* genes were derived from uncultured microorganisms and 16 were from cultured organisms, including 39935130 of *Rhodopseudomonas palustris* CGA009, 83816541 of *Salinibacter ruber* DSM 13855, 82947026 of *Magnetospirillum magneticum* AMB-1, etc (Figure 4). The results of Mantel test analysis showed abundances of *nosZ* genes were positively correlated with the concentrations of influent TN (r = 0.348, *P*<0.05) and ammonia (r = 0.338, *P*<0.01) (Table S5 in File S1).

295 *ureC* genes encoding ureases were detected, and 128 genes were shared by all samples. Unlike other nitrogen genes, most of the *ureC* genes were form cultured microorganisms and only 9 from uncultured bacteria. The results of Mantel test showed a positive correlation between *ureC* genes and the concentrations of influent TN (r = 0.463, *P*<0.01) and ammonia (r = 0.393, *P*<0.01) (Table S5 in File S1).

Only four *hzo* genes encoding hydrazine oxidoreductases were detected, including two genes (208605292 and 224712632) from *Candidatus Brocadia* sp. and two genes (224712622 and 208605290) from uncultured *Planctomycete*. No significant correlation between *hzo* genes and the concentrations of influent TN (*P*>0.05) and ammonia (*P*>0.05) (Table S5 in File S1) was observed.

### Phosphorus Cycling Genes

**Figure 5:**
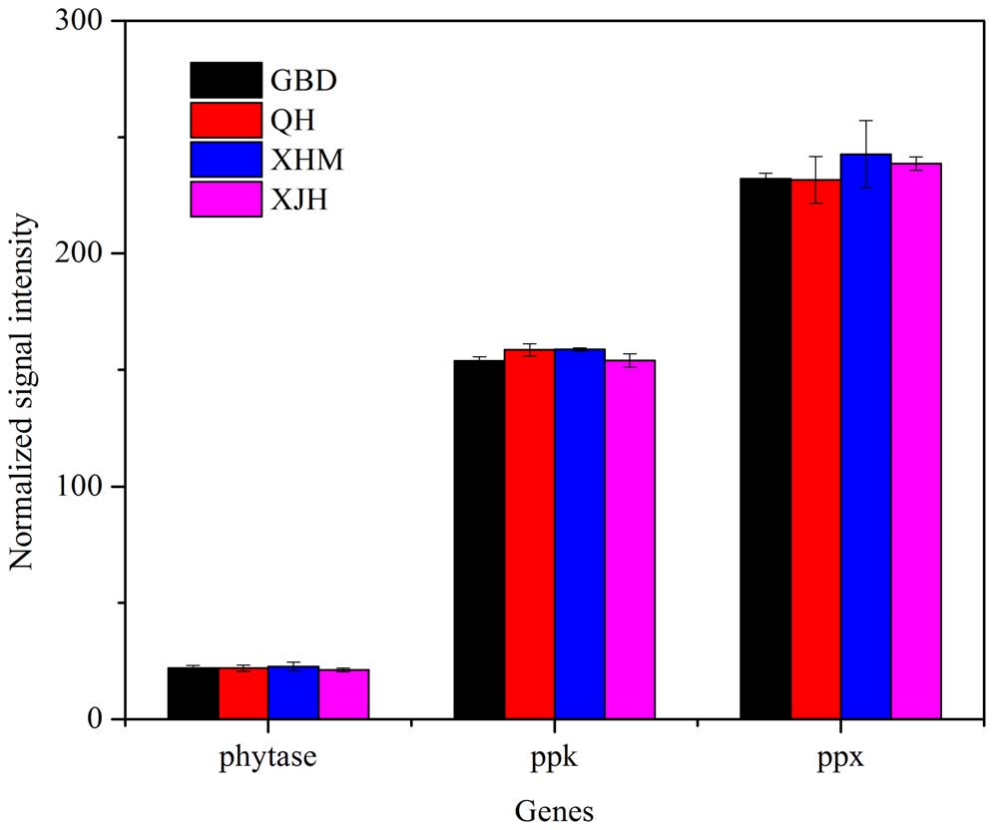
The normalized signal intensity of detected key genes involved in phosphorus cycling. The signal intensity for each functional gene was the average of the signal intensity from all the replicates. All data are presented as mean ± SE.

Phosphorus removal from WWTPs is a key factor in preventing eutrophication of surface waters. Genes encoding exopolyphosphatase (*ppx*) for inorganic polyphosphate degradation, polyphosphate kinase (*ppk*) for catalyzing the formation of polyphosphate from ATP and phytase for phytate degradation were detected among all samples (Figure 5).

A total of 90 of 246 detected *ppx* genes were shared by all samples, including genes derived from *Leptothrix cholodnii* SP-6 (171060286), *Streptomyces coelicolor* A3(2) (21221778), *Erythrobacter* sp. NAP1 (85709358). Nevertheless, there was no significant correlation between *ppx* genes and the concentrations of influent TP (Table S5 in File S1). For *ppk*, 57 of 165 detected genes were shared by all samples. Significantly positive correlation was observed between *ppk* gene abundances and the concentrations of influent TP (r = 0.242, *P*<0.05) (Table S5 in File S1). In addition, a total of 27 phytase genes were detected. Among them, 7 were shared by all samples (Figure S4 in File S1). The results of Mantel tests suggested that there was no significant correlation between phytase genes and influent TP (Table S5 in File S1).

### Sulfur Cycling Genes

**Figure 6:**
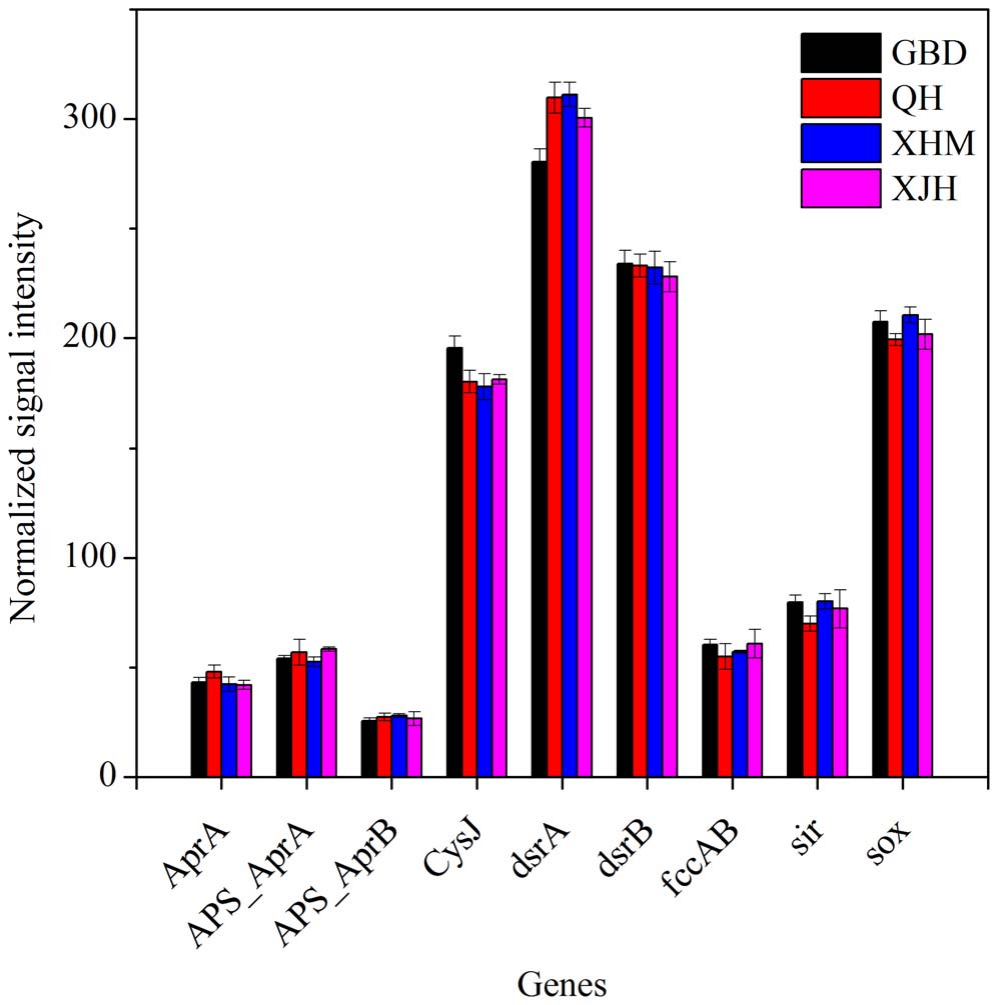
The normalized signal intensity of detected key genes involved in sulfur cycling. The signal intensity for each functional gene was the average of the signal intensity from all the replicates. All data are presented as mean ± SE.

A total of 1,241 genes involved in sulfite reductase, adenylylsulfate reductase, sulphur oxidation, sulfide oxidation were detected. The relative abundances of sulfur cycling genes were presented in Figure 6 . Among them, there were 46 *aprA* genes encoding dissimilatory adenosine-5′-phosposulfate reductase, a key enzyme involved in microbial sulfate reduction and sulfur oxidation. 15 of 22 *aprA* genes present in all 12 samples were derived from isolates (Figure S5 in File S1). In addition, 327 *dsrA* genes encoding dissimilatory sulfite reductase were detected. Of the 100 genes shared by all samples, most genes were derived from uncultured microorganisms except for 18 genes from isolates such as 107784967 of *Desulfovibrio desulfuricans*, 13992710 of *Bilophila wadsworthia* and 83573380 of *Moorella thermoacetica* ATCC 39073. For *sox* genes encoding sulfite oxidase, 77 of 205 detected genes were shared by all samples, of which most were derived from isolated microorganisms such as 56677633 of *Silicibacter pomeroyi* DSS-3, 255258527 of *Sideroxydans lithotrophicus* ES-1, 214028622 of *Ruegeria* sp. R11.

### Degradation of Organic Contaminants

**Figure 7:**
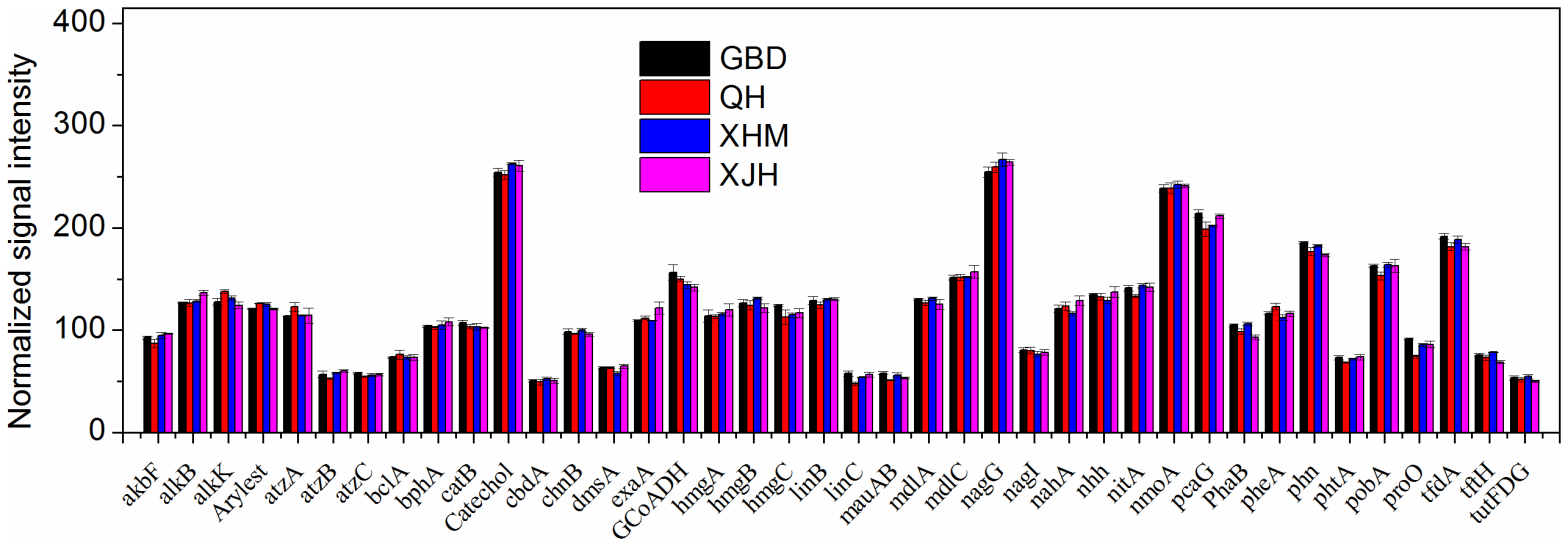
The normalized signal intensity of detected key genes involved in organic contaminant degradation. The signal intensity for each functional gene was the average of the signal intensity from all the replicates. All data are presented as mean ± SE.

Organic contamination is a concern and a lot of research has been undertaken to examine the role of microorganisms in the degradation and remediation of organic contaminants [14]. In total, 7,012 genes from 165 gene categories involved in the degradation of organic contaminants related to aromatics, chlorinated solvents, herbicides and pesticides were detected. The relative abundances of keg genes involved in organic contaminant degradation were present in Figure 7 . Among them, 24 *benAB* genes encoding benzoate 1, 2-dioxygenases were detected across all samples and 19 were present in all samples. The most abundant genes were derived from *Burkholderia xenovorans* LB400 (91779181), *Mycobacterium* sp. MCS (108768762), *Rhodococcus* sp. RHA1 (110818905).

### Metal Resistance Genes

Various heavy metals were often detected in WWTPs as a result of societal industrialization and modernization. Resistance genes of Ag, As, Cd, Co, Cr, Cu, Hg, Pb, Ni, Te and Zn were detected in this study. A substantial number (3,381) of genes involved in metal resistance were detected in all the samples, of which the vast majority was derived from isolated microorganisms. Notably, relative abundances of detected metal resistance genes were similar among activated sludges of four WWTPs (Figure S6 in File S1). In addition, the results of Mantel test showed that most of metal resistance genes, such as *chrA*, *nreB*, *copA*, *cueO*, *zntA*, *cadA* and *cadBD*, were positively correlated to the concentrations of Cr, Ni, Cu, Cu, Zn, Cd and Cd in WWTPs, respectively. In contrast, other metal resistance genes (*corC*, *cusA*, *zitB*) did not show significant correlations to the respective metal concentrations (Table S5 in File S1).

### Antibiotic Resistance

Antibiotic resistance is a growing concern worldwide as more and more pathogens develop resistance to commonly used antibiotics. 9 gene categories families related to antibiotic resistance were detected in this study, including five transporters (ATP-binding cassette, multidrug toxic compound extrusion, major facilitator superfamily, Mex, and small multidrug resistance efflux pumps), three β-lactamase genes and several genes involved in tetracycline resistance (Figure S7 in File S1). A total of 1,072 antibiotic resistance genes were detected. Of 92 tetracycline resistance genes, 39 were shared by all samples and most of them were derived from isolated microorganisms, such as 214028433 of *Ruegeria* sp. R11, 259487609 of *Aspergillus nidulans* FGSC A4, 254407454 of *Streptomyces sviceus* ATCC 29083, etc. Since antibiotic resistance is often associated with metal resistance, Mantel test was performed to examine the correlation between heavy metal resistance genes and antibiotic resistance genes. The results indicated that the whole metal resistance genes were positively correlated to the whole antibiotic resistance genes (r = 0.325, *P*<0.01).

Relationship of Environmental Factors to the Microbial Communities.

**Figure 8:**
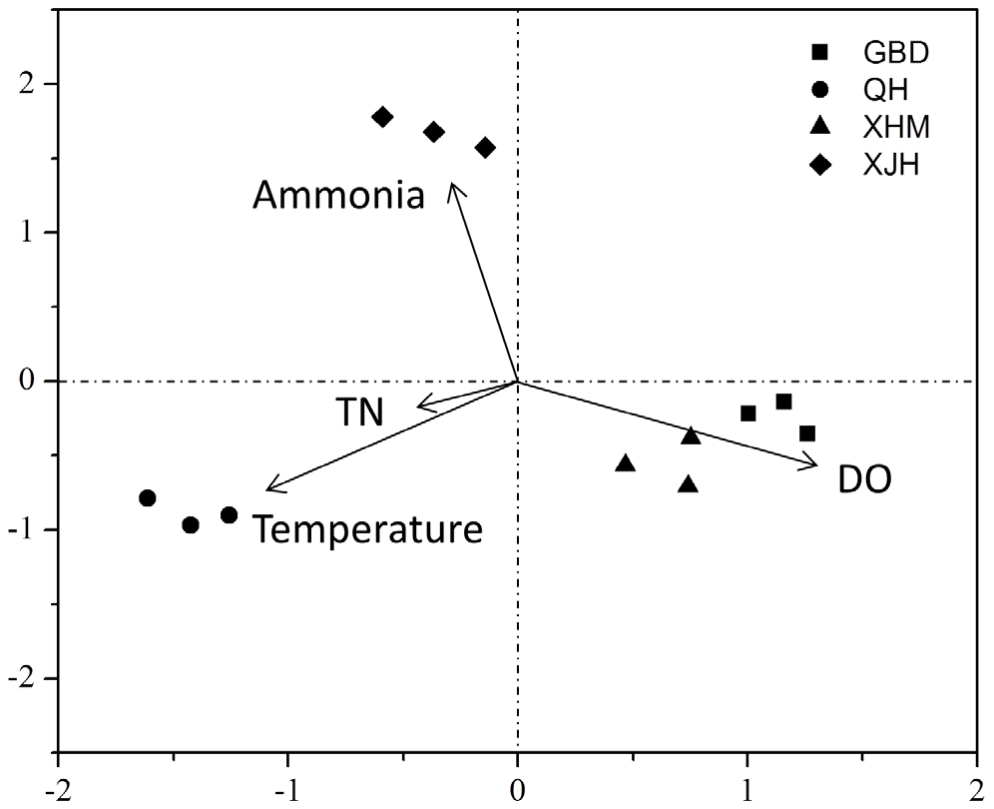
Canonical correspondence analysis (CCA) of GeoChip data and environmental variables. Environmental variables were chosen based on significance calculated from individual CCA data and variance inflation factors (VIFs).

CCA was performed to correlate environmental variables with microbial community functional structure and determine major environmental variables in shaping the microbial community structure. On the basis of variance inflation factors, four variables were selected: DO, temperature, ammonia and COD (Figure 8). The results of CCA showed that the model was significant (*P*<0.05), suggesting that four variables were major environmental factor influencing microbial community functional structure. DO showed a strong positive correlation with the first axis and a negative correlation with the second axis, while ammonia and COD loading rate showed a strong positive correlation with the second axis and a negative correlation with the first axis. Temperature showed a strong negative correlation with both the first and second axes. Microbial community functional structures in WWTPs GBD and XHM were primarily linked to DO, while QH was linked to temperature. Ammonia and COD loading rate seemed to be major factors linking to microbial community functional structures in XJH.

Variance partitioning analyses (VPA) was further performed to assess contributions of wastewater characteristics and operational parameters to microbial community variances. Wastewater characteristics included influent concentrations of COD, TN, ammonia, TP, pH and conductivity. Operational parameters consisted of DO, temperature, hydraulic retention time (HRT) and mixed liquor suspended solids (MLSS). The results showed that 53% of total variances could be explained by these variables (Figure S8 in File S1). Wastewater characteristics and operational parameters can independently explain 25% and 23% of the variation of microbial communities, respectively. Interactions among the two major components (5%) appeared to be small.

## Discussion

In this study, microbial community functional structures in activated sludges of four WWTPs were analyzed by GeoChip 4.2. A total of 29,124 functional genes involved in carbon, nitrogen, phosphorous and sulfur cycles, metal resistance, antibiotic resistance and organic contaminant degradation were detected. It is reasonable to expect that the high diversity of functional genes can increase the functional redundancy, which is ensured by the presence of a pool of species able to perform the same ecological function. That is to say, many species share similar traits in activated sludge, therefore the loss of a few species has little impact on the system performance. Functional redundancy of microbial community in activated sludge is important to ensure the system stability when individual species are lost due to environmental changes, such as significant changes in the influent wastewater composition [4].

For individual gene category, such as *egl*, *amyA*, *lip*, *nirS*, *nirK*, *nosZ*, *ureC*, *ppx*, *ppk*, *aprA*, *dsrA*, *sox* and *benAB*, high diversity were detected. For example, 110 *nosZ* genes were detected in this study. *nosZ* gene encodes nitrous oxide reductase that is the only enzyme to mediate the conversion of N_2_O to N_2_ during denitrification process, which makes the *nosZ* gene an important molecular marker to trace complete denitrification [28]. This high diversity of *nosZ* genes ensures the presence of different species with similar function mediating the conversion of N_2_O to N_2_, which could lead to a more stable denitrification in WWTPs. Similarly, the high diversity of *egl*, *amyA*, etc. can increase the functional redundancy, and ensure the stable operation of plants.

GeoChip data revealed high similarities among microbial community functional structures within activated sludge of four WWTPs as measured by diversity indices and overlapped genes. This can be partly explained by the fact that all the WWTPs studied were located in the same city (Beijing), used to treat similar domestic wastewater, and operated with the same anaerobic/anoxic/aerobic (A^2^O) process of similar operating parameters (COD and TN loading rate and MLSS concentration). For each gene category such as *egl, amyA*, *lip*, *nirS*, *nirK*, *nosZ*, *ureC*, *ppx*, *ppk*, *aprA*, *dsrA*, *sox*, *benAB*, there were a number of microorganisms commonly shared by all 12 samples, suggesting that there could be a core microbial community in activated sludge of four WWTPs. This agrees with the findings of zhang et al.[29], who suggested, based on pyrosequencing surveys of 14 WWTPs, that some core populations were shared by multiple samples. Similar results were also reported by other studies [30], [31].

Wastewater contains a broad spectrum of organic contaminants resulting from the mixing of wastewaters from different sources. Starch, cellulose, chitin and lignin could be the major carbon and energy sources for various microbial populations. Thus, we hypothesized that microbial communities would be abundant in functional genes involved in starch, cellulose, chitin and lignin. Our GeoChip data validated the hypothesis by evidence of detecting a wide variety of these genes across all samples such as *egl*, *amyA*, *lip*, xylanase genes, etc. Also, in WWTPs, biological nitrification coupled with denitrification is widely used to remove ammonia from wastewater. Nitrification is the main supply pathway of NO_3_^−^ for denitrification, and thus it is reasonable to expect the genes involved in nitrogen cycle would be tightly linked with NH_4_^+^ and NO_3_^−^ concentration. The results of Mantel test supported this expectation (Table S5 in File S1).

Understanding the factors that shape microbial community structure in WWTPs could potentially enhance treatment performance and control [11]. Of the 9 operational and environmental variables tested in this study, CCA ordination analysis indicated that DO was an important variable influencing microbial community structures. This agreed with the findings of Wells *et al.* [32], which suggested that DO was one of the most influential variables on microbial community structures of ammonia-oxidizing bacteria (AOB). Similar results have also been obtained in several lab-scale bioreactors by Park *et al.* [33].

Our results indicated that water temperature was also significantly linked to the microbial community structures in WWTPs, which was consistent with previous observations that water temperature was an important factor in shaping the microbial community structures. Ebrahimi *et al.* [34] found that, in two sequencing batch reactors (SBR) operated at 20°C, 30°C and 35°C, bacterial richness and diversity were clearly altered. In a membrane bioreactor treating domestic sewage, Ebie *et al.* [35] revealed shifts in AOB lineages in response to temperature changes by using denaturing gradient gel electrophoresis. Several other studies also demonstrated that temperature imposed a strong selective pressure on microbial communities of activated sludge [36]–[38].

In addition to DO and water temperature, ammonia concentrations within WWTPs were strongly and significantly linked to microbial community functional structures. Some previous studies have demonstrated the importance of ammonia concentration within bioreactor. In three gravel biofilters treating saline wastewater, Gregory *et al.* [39] observed that increasing ammonia concentration significantly influenced community structures of total bacteria and AOB. Miura *et al.* [40] found that ammonia concentration was an important structuring factor based on the DGGE analysis of AOB communities in four pilot-scale wastewater treatment systems with different ammonia concentrations.

The importance of COD loading rate for microbial community structures has been shown by previous studies. Pholchan *et al.* [41] reported that in laboratory-scale activated sludge reactors, high COD loading rate resulted in an increase and decrease of community diversity of total bacteria and AOB, respectively. In addition, Zhou *et al.* [42] showed that organic carbon (equivalent to COD loading rates) significantly affected microbial diversity in soil.

VPA was performed to attribute variations observed in microbial community functional structures to wastewater characteristics and operational parameters. VPA results showed that the deterministic wastewater characteristics and operational factors explained 53% of variances of microbial community functional structures, thus 47% of the variance was attributed to unknown factors. Some of them may result from unknown environmental variables or additional factors such as stochastic dispersal and immigration processes [43], [44], protozoan grazing [45], phage predation [46] and chaotic behavior [47], [48], which may play an influential role in shaping microbial communities in WWTPs.

Like other microarray technologies, GeoChip only detects genes that are represented on the array. Therefore, GeoChip 4.2 used in this study may not cover all of functional genes of microbial communities, though it contains as many as 83,992 probes targeting 152,414 genes involved in major microbial biogeochemical processes. Also, in our study, the DNA rather than cDNA from RNA was used for GeoChip hybridization. It is argued that DNA-based community analysis detects microbes irrespective of their viability or metabolic activity. Furthermore, DNA could persist extracellularly in environments after cell death, and it could result in biased population profiles [49].

In conclusion, GeoChip 4.2 was used to evaluate the microbial functional genes of activated sludge in four WWTPs. Our results revealed high similarities of microbial community functional structures among activated sludges of four WWTPs as measured by diversity indices and overlapped genes. CCA analysis indicated that microbial community functional structures were highly correlated to the water temperature, DO, ammonia concentrations and COD loading rate. Finally, a total of 53% of microbial community variations from GeoChip data were explainable by wastewater characteristics and operational parameters.

## Supporting Information

File S1 **This includes Tables S1–S5 and Figures S1–S8.** Table S1 Summary of overall operating parameters of four WWTPs. Table S2 Metal concentrations within the four WWTPs. Table S3 Phylogenetic classification based on GeoChip data. Table S4 Numbers of detected genes involved in carbon cycling and total gene numbers present on GeoChip. Table S5 The relationship of different gene categories to related environmental variables revealed by Mantel test. Figure S1 Hierarchical cluster analysis of egl genes. Figure S2 The hierarchical cluster analysis of Xylanase genes. Figure S3 The hierarchical cluster analysis of lip genes. Figure S4 Hierarchical cluster analysis of phytase genes. Figure S5 The hierarchical cluster analysis of aprA genes. Figure S6 The normalized signal intensity of detected key genes involved in metal resistance. Figure S7 The normalized signal intensity of detected key genes involved in Antibiotic resistance. Figure S8 Variation partitioning analysis of microbial diversity explained by wastewater characteristics (W) and operational parameters (O).
(DOC)
