# Supplementary material for: Microbial Community Functional Structures in Wastewater Treatment Plants as Characterized by GeoChip
Source: PLoS One. 2014 Mar 26;9(3):e93422. doi: 10.1371/journal.pone.0093422 (PMC3966879; doi:10.1371/journal.pone.0093422)
Supplement: File S1 — This includes Tables S1–S5 and Figures S1–S8. Table S1 Summary of overall operating parameters of four WWTPs. Table S2 Metal concentrations within the four WWTPs. Table S3 Phylogenetic classification based on GeoChip data. Table S4 Numbers of detected genes involved in carbon cycling and total gene numbers present on GeoChip. Table S5 The relationship of different gene categories to related environmental variables revealed by Mantel test. Figure S1 Hierarchical cluster analysis of egl genes. Figure S2 The hierarchical cluster analysis of Xylanase genes. Figure S3 The hierarchical cluster analysis of lip genes. Figure S4 Hierarchical cluster analysis of phytase genes. Figure S5 The hierarchical cluster analysis of aprA genes. Figure S6 The normalized signal intensity of detected key genes involved in metal resistance. Figure S7 The normalized signal intensity of detected key genes involved in Antibiotic resistance. Figure S8 Variation partitioning analysis of microbial diversity explained by wastewater characteristics (W) and operational parameters (O). (DOC) [file pone.0093422.s001.doc]

**Supporting Information**

1. **SUPPORTING TABLES**

Table S1 Summary of overall operating parameters of four WWTPs

Table S2 Metal concentrations within the four WWTPs

Table S3 Phylogenetic classification based on GeoChip data

Table S4 Numbers of detected genes involved in carbon cycling and total gene numbers present on GeoChip

Table S5 The relationship of different gene categories to related environmental variables revealed by Mantel test

1. **SUPPORTING FIGURES**

Figure S1 Hierarchical cluster analysis of egl genes.

Figure S2 The hierarchical cluster analysis of Xylanase genes.

Figure S3 The hierarchical cluster analysis of *lip* genes.

Figure S4 Hierarchical cluster analysis of phytase genes.

Figure S5 The hierarchical cluster analysis of *aprA* genes.

Figure S6 The normalized signal intensity of detected key genes involved in metal resistance.

Figure S7 The normalized signal intensity of detected key genes involved in Antibiotic resistance.

Figure S8 Variation partitioning analysis of microbial diversity explained by wastewater characteristics (W) and operational parameters (O).

Table S1. Summary of overall operating parameters of four WWTPs ^a^

| WWTPs | COD  (mg L^-1^) | COD removal rate (%) | COD loading rate  kg (m^3^·d)^-1^ | TN  (mg L^-1^) | TN removal rate (%) | TN  loading rate  kg(m^3^·d)^-1^ | Ammonia  (mg L^-1^) | TP  (mg L^-1^) | pH | Conductivity  (μs cm^-1^) | DO  (mg L^-1^) | Temperature  (°C) | MLSS  (mg L^-1^) | HRT  (h) |
| --- | --- | --- | --- | --- | --- | --- | --- | --- | --- | --- | --- | --- | --- | --- |
| GBD | 452 | 94 | 0.34 | 58 | 69 | 0.04 | 52 | 6.0 | 6.9 | 1390 | 1.4 | 20.7 | 3500 | 9 |
| QH | 437 | 90 | 0.36 | 64 | 71 | 0.05 | 58 | 6.1 | 7.2 | 824 | 3.8 | 23.1 | 3200 | 9 |
| XHM | 443 | 91 | 0.35 | 46 | 68 | 0.04 | 36 | 6.8 | 7.3 | 1690 | 1.1 | 26.0 | 3400 | 9 |
| XJH | 257 | 93 | 0.27 | 48 | 75 | 0.05 | 42 | 6.1 | 7.2 | 1020 | 2.9 | 19.8 | 3800 | 6 |

^a^All the four WWTPs were full-scale, and operated with anaerobic/anoxic/aerobic (A^2^O) process.

Table S2. Metal concentrations within the four WWTP

| WWTPs | Cr  (mg L^-1^) | Co  (mg L^-1^) | Ni  (mg L^-1^) | Cu  (mg L^-1^) | Zn  (mg L^-1^) | Cd  (mg L^-1^) |
| --- | --- | --- | --- | --- | --- | --- |
| GBD | 9.57 | 0.64 | 9.70 | 2.33 | 39.91 | 0.03 |
| QH | 4.67 | 0.51 | 3.42 | 2.38 | 35.98 | 0.02 |
| XHM | 9.57 | 0.61 | 7.22 | 2.80 | 46.23 | 0.02 |
| XJH | 9.94 | 0.61 | 6.47 | 1.55 | 29.64 | 0.02 |

Table S3 Phylogenetic classification based on GeoChip data^a^

| Phylogenetic groups | GBD_1 | GBD_2 | GBD_3 | QH_1 | QH_2 | QH_3 | XHM_1 | XHM_2 | XHM_3 | XJH_1 | XJH_2 | XJH_3 |
| --- | --- | --- | --- | --- | --- | --- | --- | --- | --- | --- | --- | --- |
| *Archaea* | 402 | 400 | 401 | 364 | 386 | 381 | 415 | 407 | 410 | 342 | 372 | 368 |
| *Crenarchaeota* | 58 | 60 | 56 | 59 | 61 | 56 | 60 | 63 | 61 | 51 | 60 | 58 |
| *Euryarchaeota* | 339 | 335 | 340 | 299 | 319 | 320 | 349 | 338 | 342 | 287 | 306 | 304 |
| *Korarchaeota* | 2 | 2 | 2 | 3 | 3 | 2 | 3 | 4 | 4 | 3 | 3 | 3 |
| *Thaumarchaeota* | 3 | 3 | 3 | 3 | 3 | 3 | 3 | 2 | 3 | 1 | 3 | 3 |
| *Bacteria* | 19038 | 18121 | 18723 | 15699 | 16473 | 16718 | 18794 | 18372 | 19518 | 15190 | 16808 | 16724 |
| *Acidobacteria* | 95 | 94 | 95 | 74 | 87 | 86 | 88 | 92 | 96 | 74 | 78 | 81 |
| *Actinobacteria* | 3686 | 3500 | 3624 | 2961 | 3091 | 3214 | 3588 | 3494 | 3730 | 2946 | 3251 | 3251 |
| *Aquificae* | 18 | 19 | 16 | 21 | 20 | 15 | 17 | 18 | 16 | 21 | 22 | 20 |
| *Bacteroidetes* | 309 | 304 | 309 | 306 | 314 | 299 | 327 | 317 | 342 | 274 | 290 | 292 |
| *Candidatus_Poribacteria* | 2 | 2 | 2 | 1 | 1 | 1 | 2 | 2 | 2 | 2 | 2 | 2 |
| *Chlamydiae* | 6 | 8 | 8 | 8 | 8 | 6 | 7 | 8 | 7 | 6 | 9 | 10 |
| *Chlorobi* | 111 | 106 | 103 | 88 | 96 | 95 | 114 | 112 | 122 | 80 | 95 | 97 |
| *Chloroflexi* | 262 | 252 | 258 | 234 | 244 | 244 | 262 | 262 | 280 | 213 | 241 | 237 |
| *Cyanobacteria* | 445 | 425 | 429 | 422 | 434 | 428 | 463 | 452 | 460 | 392 | 418 | 422 |
| *Deferribacteres* | 2 | 3 | 2 | 2 | 2 | 2 | 3 | 3 | 4 | 2 | 2 | 2 |
| *Deinococcus-Thermus* | 183 | 174 | 172 | 167 | 173 | 164 | 186 | 188 | 188 | 161 | 173 | 173 |
| *Dictyoglomi* | 2 | 2 | 1 | 3 | 3 | 2 | 2 | 2 | 2 | 1 | 1 | 1 |
| *Elusimicrobia* | 3 | 3 | 3 | 5 | 5 | 3 | 3 | 3 | 3 | 5 | 5 | 4 |
| *Fibrobacteres* | 6 | 6 | 6 | 7 | 6 | 5 | 8 | 6 | 8 | 6 | 5 | 6 |
| *Firmicutes* | 1090 | 1070 | 1073 | 1022 | 1045 | 987 | 1093 | 1065 | 1138 | 951 | 1007 | 994 |
| *Fusobacteria* | 3 | 3 | 3 | 4 | 4 | 3 | 3 | 3 | 3 | 4 | 4 | 4 |
| *Gemmatimonadetes* | 22 | 22 | 23 | 18 | 17 | 18 | 26 | 25 | 28 | 18 | 20 | 22 |
| *Lentisphaerae* | 24 | 21 | 22 | 17 | 21 | 20 | 22 | 21 | 23 | 16 | 17 | 16 |
| *Nitrospirae* | 12 | 12 | 12 | 11 | 10 | 9 | 10 | 9 | 11 | 9 | 10 | 8 |
| *Planctomycetes* | 119 | 113 | 118 | 102 | 111 | 106 | 117 | 112 | 116 | 96 | 108 | 108 |
| *α-Proteobacteria* | 4776 | 4458 | 4706 | 3755 | 3958 | 4096 | 4624 | 4525 | 4818 | 3699 | 4113 | 4073 |
| *β-Proteobacteria* | 3141 | 2939 | 3085 | 2408 | 2565 | 2648 | 3024 | 2973 | 3180 | 2372 | 2689 | 2673 |
| *γ-Proteobacteria* | 3251 | 3147 | 3219 | 2753 | 2890 | 2921 | 3275 | 3200 | 3392 | 2590 | 2902 | 2872 |
| *δ-Proteobacteria* | 1113 | 1092 | 1078 | 964 | 1017 | 1007 | 1143 | 1110 | 1160 | 938 | 1005 | 1008 |
| *ε-Proteobacteria* | 62 | 62 | 62 | 69 | 66 | 62 | 68 | 68 | 71 | 56 | 59 | 66 |
| *ζ-Proteobacteria* | 7 | 8 | 8 | 9 | 8 | 7 | 10 | 8 | 8 | 7 | 8 | 7 |
| *Spirochaetes* | 15 | 14 | 16 | 16 | 22 | 19 | 21 | 17 | 19 | 17 | 17 | 18 |
| *Synergistetes* | 17 | 14 | 15 | 20 | 20 | 18 | 19 | 19 | 19 | 15 | 18 | 17 |
| *Tenericutes* | 5 | 5 | 4 | 6 | 6 | 5 | 6 | 6 | 5 | 6 | 6 | 5 |
| *Thermodesulfobacteria* | 2 | 2 | 2 | 2 | 2 | 2 | 2 | 2 | 2 | 2 | 2 | 2 |
| *Thermotogae* | 13 | 14 | 14 | 18 | 17 | 13 | 18 | 14 | 17 | 12 | 13 | 14 |
| *Verrucomicrobia* | 190 | 183 | 190 | 168 | 173 | 176 | 197 | 195 | 201 | 161 | 175 | 178 |
| *Eukaryote* | 1924 | 1854 | 1819 | 1818 | 1849 | 1840 | 2041 | 1952 | 2086 | 1588 | 1756 | 1773 |
| *Arthropoda* | 3 | 2 | 3 | 2 | 2 | 1 | 2 | 1 | 2 | 1 | 1 | 1 |
| *Ascomycota* | 1525 | 1467 | 1448 | 1456 | 1482 | 1479 | 1628 | 1556 | 1665 | 1260 | 1393 | 1406 |
| *Bacillariophyta* | 3 | 2 | 2 | 1 | 1 | 2 | 3 | 2 | 3 | 2 | 1 | 2 |
| *Basidiomycota* | 372 | 359 | 344 | 338 | 341 | 340 | 386 | 373 | 397 | 310 | 341 | 344 |
| *Chlorophyta* | 2 | 2 | 1 | 1 | 1 | 1 | 1 | 2 | 1 | 1 | 1 | 1 |
| *Chordata* | 2 | 3 | 3 | 2 | 2 | 1 | 3 | 4 | 2 | 2 | 2 | 3 |
| *Echinodermata* | 2 | 3 | 3 | 4 | 3 | 2 | 3 | 2 | 2 | 2 | 3 | 3 |
| *Glomeromycota* | 1 | 2 | 1 | 2 | 2 | 1 | 2 | 2 | 2 | 1 | 1 | 1 |
| *Microsporidia* | 3 | 3 | 3 | 2 | 2 | 2 | 2 | 1 | 2 | 2 | 2 | 2 |
| *Phaeophyceae* | 1 | 1 | 1 | 1 | 1 | 1 | 1 | 1 | 1 | 0 | 1 | 1 |
| *Streptophyta* | 10 | 10 | 10 | 9 | 12 | 10 | 10 | 8 | 9 | 7 | 10 | 9 |

^a^All the numbers are functional gene number detected by GeoChip. Functional genes were grouped based on phylogenetic markers on the GeoChip 4.2. GBD, QH, XHM and XJH are four WWTPs we studied. Each plant has three replicate samples indicated with “_1”, “_2”, and “_3”.

Table S4 Numbers of detected genes involved in carbon cycling and total gene numbers present on GeoChip

| Functional gene | GBD_1 | GBD_2 | GBD_3 | QH_1 | QH_2 | QH_3 | XHM_1 | XHM_2 | XHM_3 | XJH_1 | XJH_2 | XJH_3 | GeoChip^a^ |
| --- | --- | --- | --- | --- | --- | --- | --- | --- | --- | --- | --- | --- | --- |
| Alpha Amylase | 239 | 275 | 265 | 304 | 285 | 305 | 269 | 277 | 275 | 295 | 281 | 288 | 1194 |
| Cyclomaltodextrinase | 60 | 64 | 68 | 69 | 77 | 75 | 63 | 66 | 61 | 74 | 71 | 71 | 480 |
| Glucoamylase | 32 | 33 | 31 | 33 | 36 | 33 | 35 | 35 | 35 | 34 | 35 | 36 | 148 |
| Neopullulanase | 40 | 41 | 38 | 46 | 43 | 46 | 44 | 42 | 44 | 43 | 41 | 40 | 394 |
| Pullulanase | 54 | 61 | 59 | 66 | 63 | 66 | 57 | 58 | 61 | 65 | 64 | 64 | 244 |
| Cellobiase | 53 | 55 | 60 | 61 | 66 | 65 | 54 | 56 | 56 | 65 | 64 | 62 | 248 |
| Endoglucanase | 48 | 47 | 48 | 51 | 53 | 53 | 51 | 47 | 49 | 55 | 53 | 49 | 187 |
| Exoglucanase | 32 | 34 | 33 | 41 | 35 | 40 | 41 | 40 | 40 | 37 | 33 | 36 | 213 |
| Arabinofuranosidase(fungi) | 104 | 122 | 117 | 133 | 125 | 139 | 113 | 113 | 119 | 131 | 124 | 125 | 543 |
| Arabinofuranosidase | 104 | 122 | 117 | 133 | 125 | 139 | 113 | 113 | 119 | 131 | 124 | 125 | 543 |
| Mannanase | 32 | 37 | 37 | 42 | 37 | 45 | 36 | 36 | 36 | 38 | 37 | 36 | 183 |
| Xylose isomerase | 62 | 77 | 74 | 77 | 77 | 80 | 65 | 73 | 74 | 87 | 84 | 83 | 341 |
| Xylanase | 29 | 30 | 31 | 38 | 35 | 37 | 31 | 36 | 33 | 37 | 33 | 34 | 152 |
| Acetylglucosaminidase | 107 | 111 | 108 | 127 | 119 | 129 | 106 | 112 | 109 | 121 | 116 | 119 | 556 |
| Endochitinase | 161 | 173 | 172 | 195 | 187 | 197 | 166 | 173 | 166 | 185 | 182 | 174 | 796 |
| Exochitinase | 16 | 17 | 16 | 21 | 22 | 24 | 17 | 18 | 18 | 23 | 22 | 22 | 122 |
| Glyoxal oxidase | 36 | 36 | 36 | 40 | 39 | 39 | 36 | 35 | 34 | 39 | 36 | 34 | 147 |
| Lignin peroxidase | 20 | 21 | 22 | 23 | 25 | 26 | 20 | 19 | 20 | 24 | 22 | 22 | 110 |
| Manganese peroxidase | 16 | 18 | 19 | 20 | 20 | 22 | 13 | 16 | 16 | 20 | 19 | 17 | 112 |
| Phenol oxidase | 113 | 123 | 122 | 137 | 125 | 136 | 127 | 123 | 123 | 134 | 129 | 134 | 622 |

^a^ The total number of genes on the GeoChip 4.2.

Table S5 The relationship of different gene categories to related environmental variables revealed by Mantel test ^a^

| Gene category | Environmental variable | 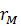^b^ | *P* |
| --- | --- | --- | --- |
| *nirS* | Influent TN | 0.4892 | **0.004** |
|  | Influent ammonia | 0.4980 | **0.001** |
| *nirK* | Influent TN | 0.4212 | **0.003** |
|  | Influent ammonia | 0.3579 | **0.012** |
| *nosZ* | Influent TN | 0.3484 | **0.020** |
|  | Influent ammonia | 0.3384 | **0.013** |
| *ureC* | Influent TN | 0.4631 | **0.005** |
|  | Influent ammonia | 0.3927 | **0.007** |
| *hzo* | Influent TN | -0.0757 | 0.733 |
|  | Influent ammonia | -0.0979 | 0.789 |
| *ppx* | Influent TP | 0.1006 | 0.252 |
| *ppk* | Influent TP | 0.2423 | **0.043** |
| phytase gene | Influent TP | 0.2423 | 0.054 |
| *chrA* | Cr | 0.6458 | **0.001** |
| *corC* | Co | 0.1672 | 0.185 |
| *nreB* | Ni | 0.3326 | **0.026** |
| *copA* | Cu | 0.2929 | **0.021** |
| *cueO* | Cu | 0.3414 | **0.010** |
| *cusA* | Cu | 0.1269 | 0.205 |
| *zntA* | Zn | 0.4361 | **0.005** |
| *zitB* | Zn | 0.1674 | 0.128 |
| *cadA* | Cd | 0.3524 | **0.022** |
| *cadBD* | Cd | 0.3524 | **0.030** |

^a^ The signal intensity of different functional genes among 12 samples was used as the first matrix; normalized related environmental variables were used as the second matrix. Boldface values indicate significant P values (P<0.05).

^b^ *r_M_*, Mantel’s correlation coefficient.


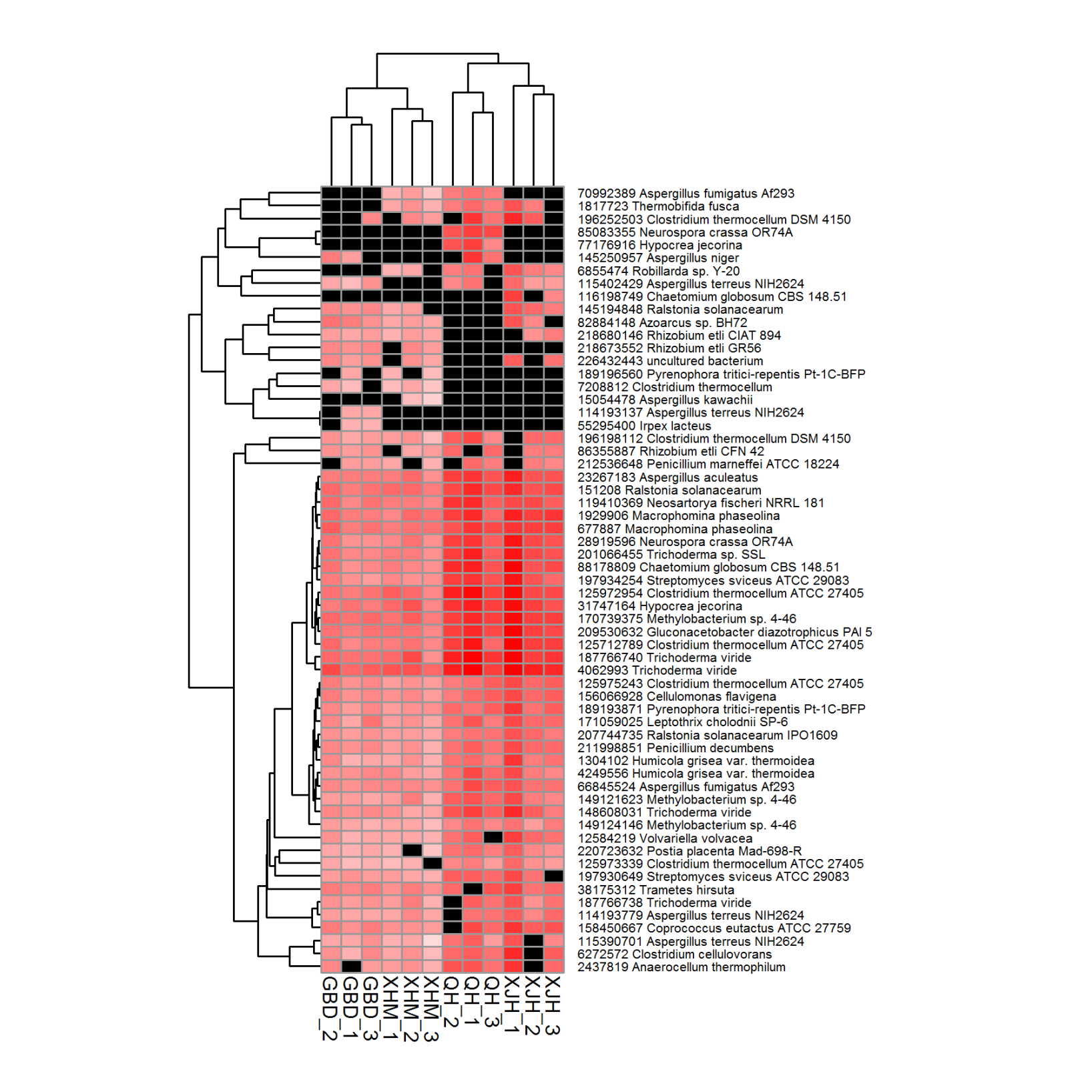


Figure S1 Hierarchical cluster analysis of *egl* genes encoding endoglucanases. The protein id number and its derived organism for each gene are indicated. The color intensity of each panel shows the normalized signal intensity of individual genes, referring to color key at the top right..


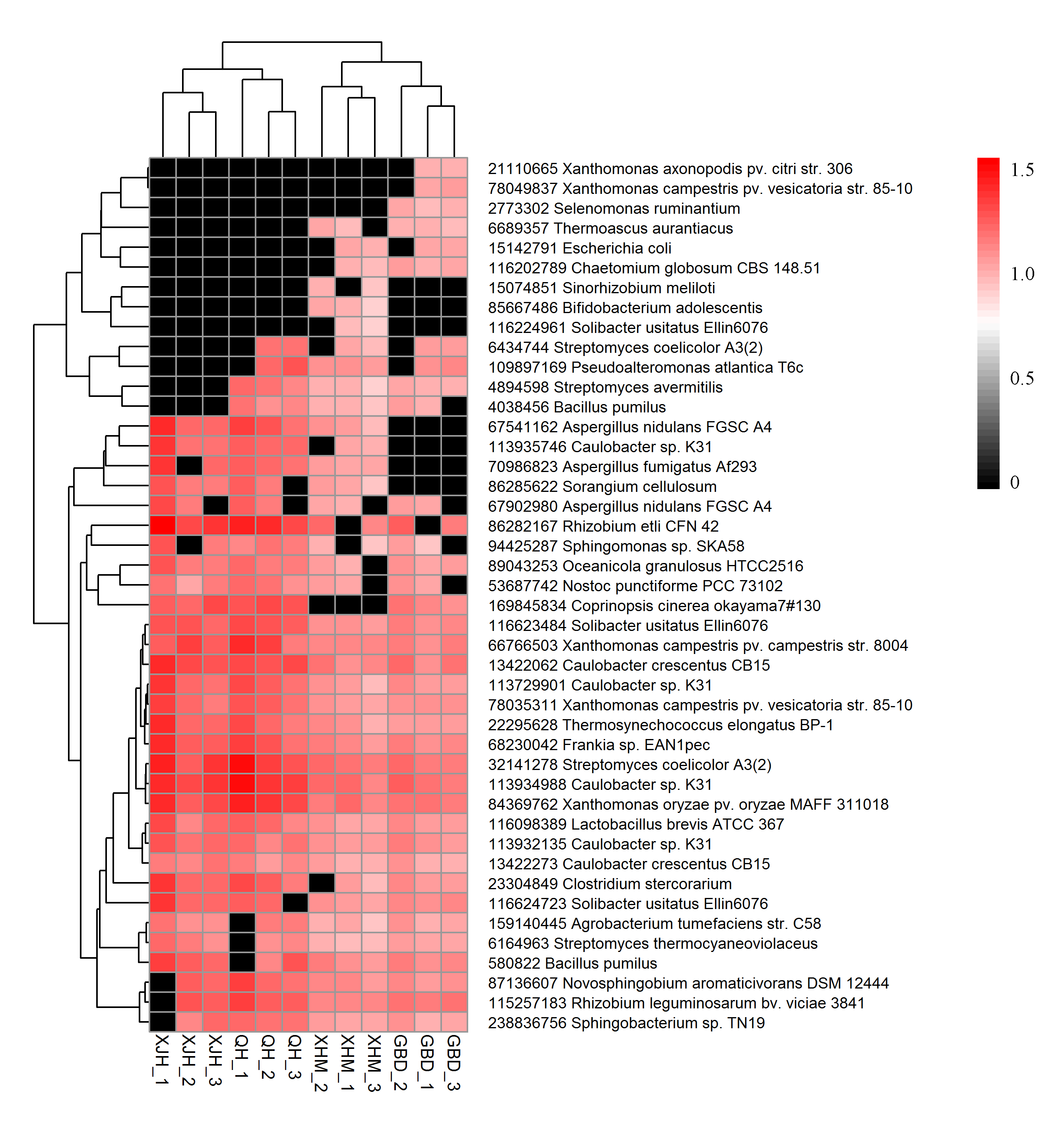


Figure S2 The hierarchical cluster analysis of *Xylanase* genes. The protein id number and its derived organism for each gene are indicated. The color intensity in each panel represents normalized signal intensity of individual genes in a sample, referring to color key at the top right.


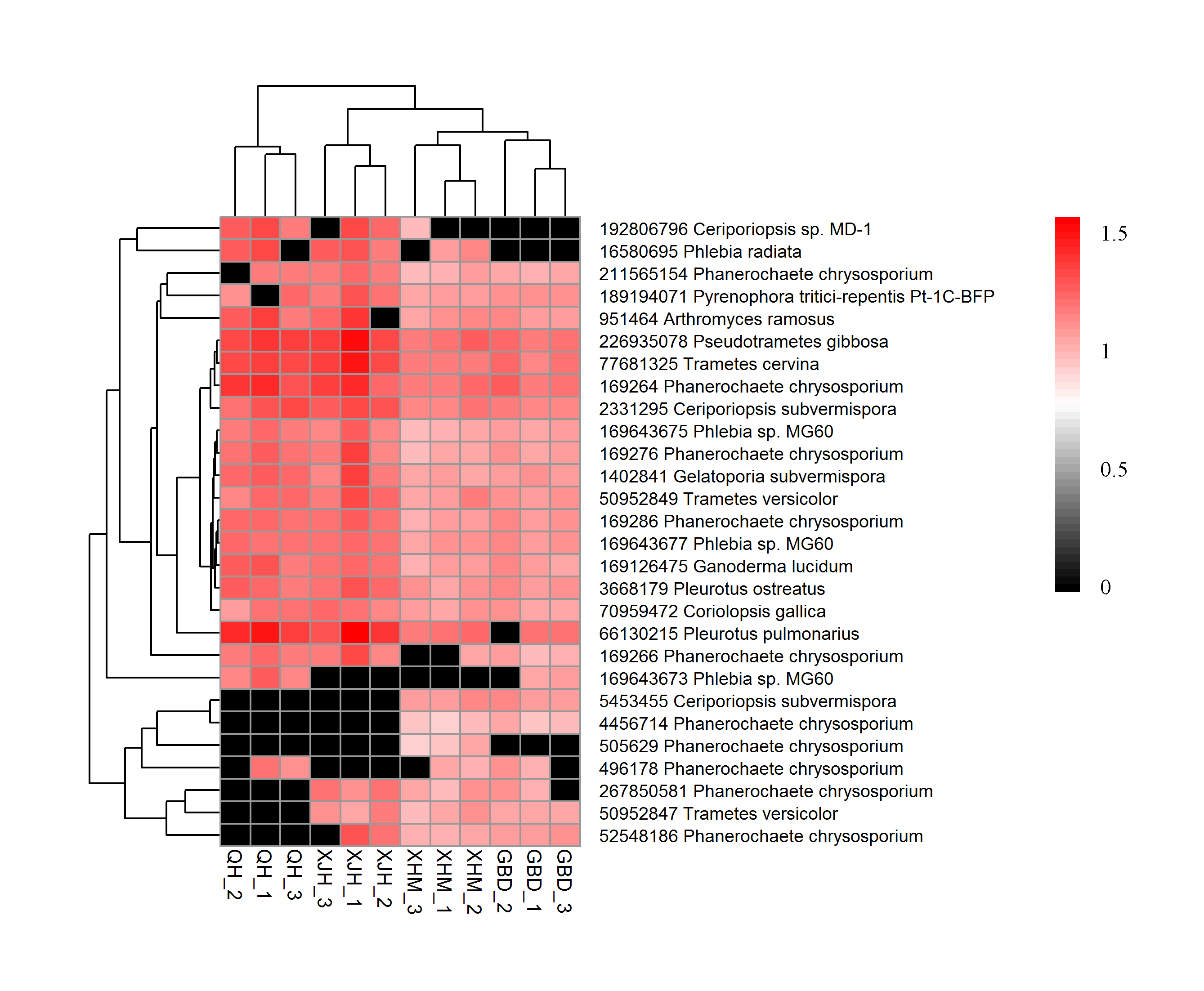


Figure S3 The hierarchical cluster analysis of *lip* genes. The protein id number and its derived organism for each gene are indicated. The color intensity in each panel represents normalized signal intensity of individual genes in a sample, referring to color key at the top right.


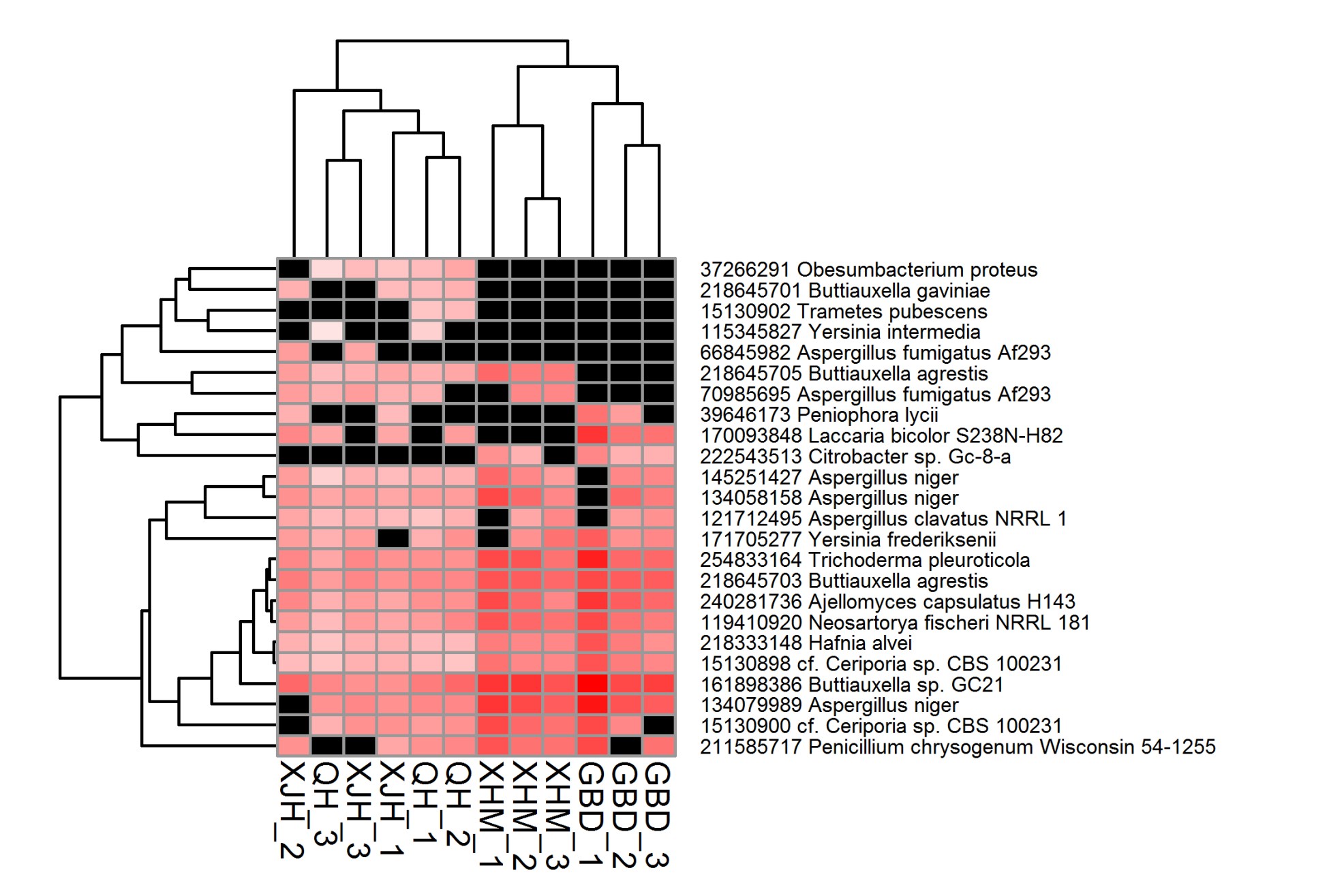


Figure S4 Hierarchical cluster analysis of phytase genes. The protein id number and its derived organism for each gene are indicated. The color intensity of each panel shows the normalized signal intensity of individual genes, referring to color key at the top right.


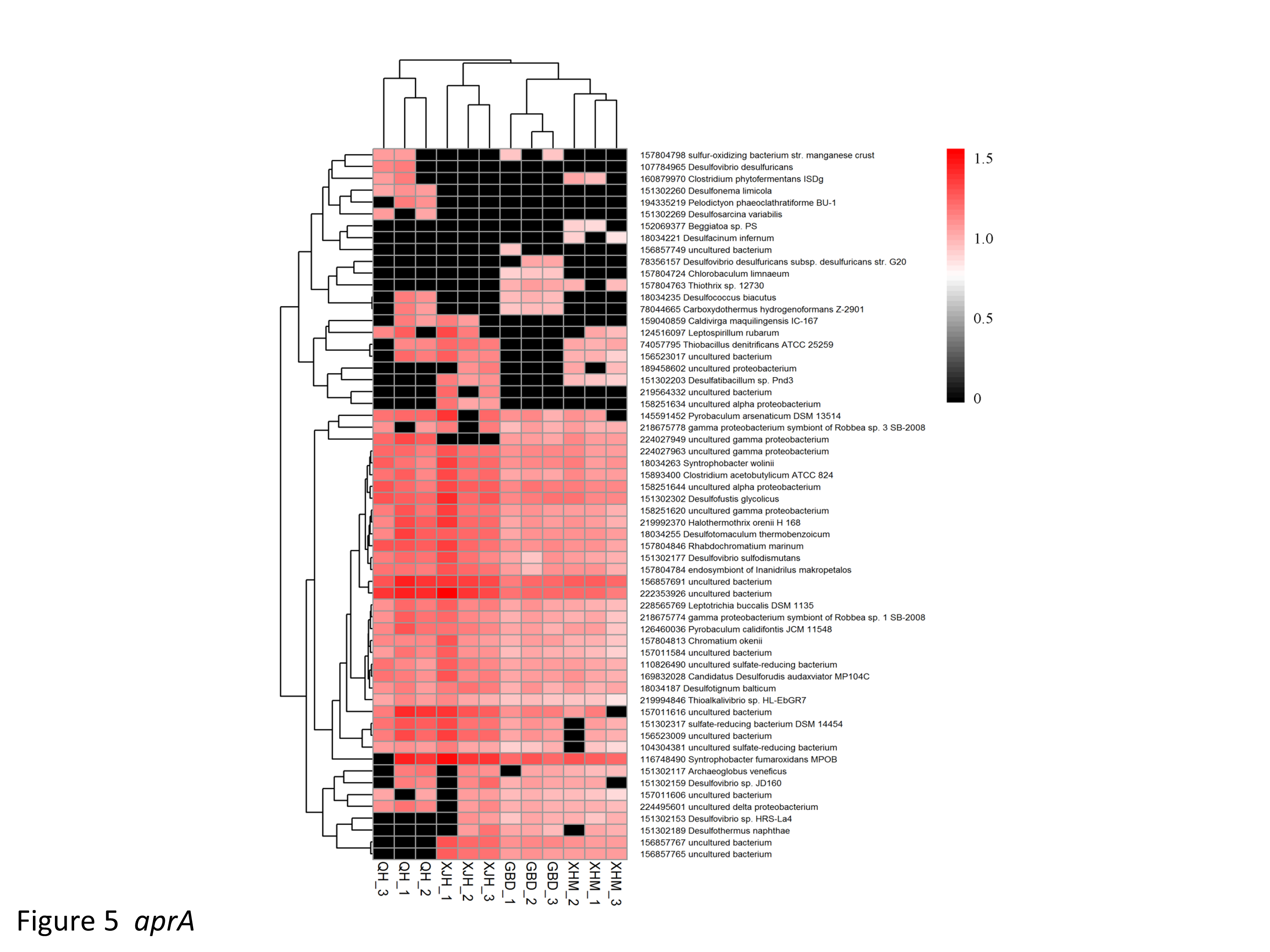


Figure S5 Hierarchical cluster analysis of *aprA* genes encoding dissimilatory adenosine-5'-phosposulfate reductases. The protein id number and its derived organism for each gene are indicated. The color intensity of each panel shows the normalized signal intensity of individual genes in a sample, referring to color key at the top right.


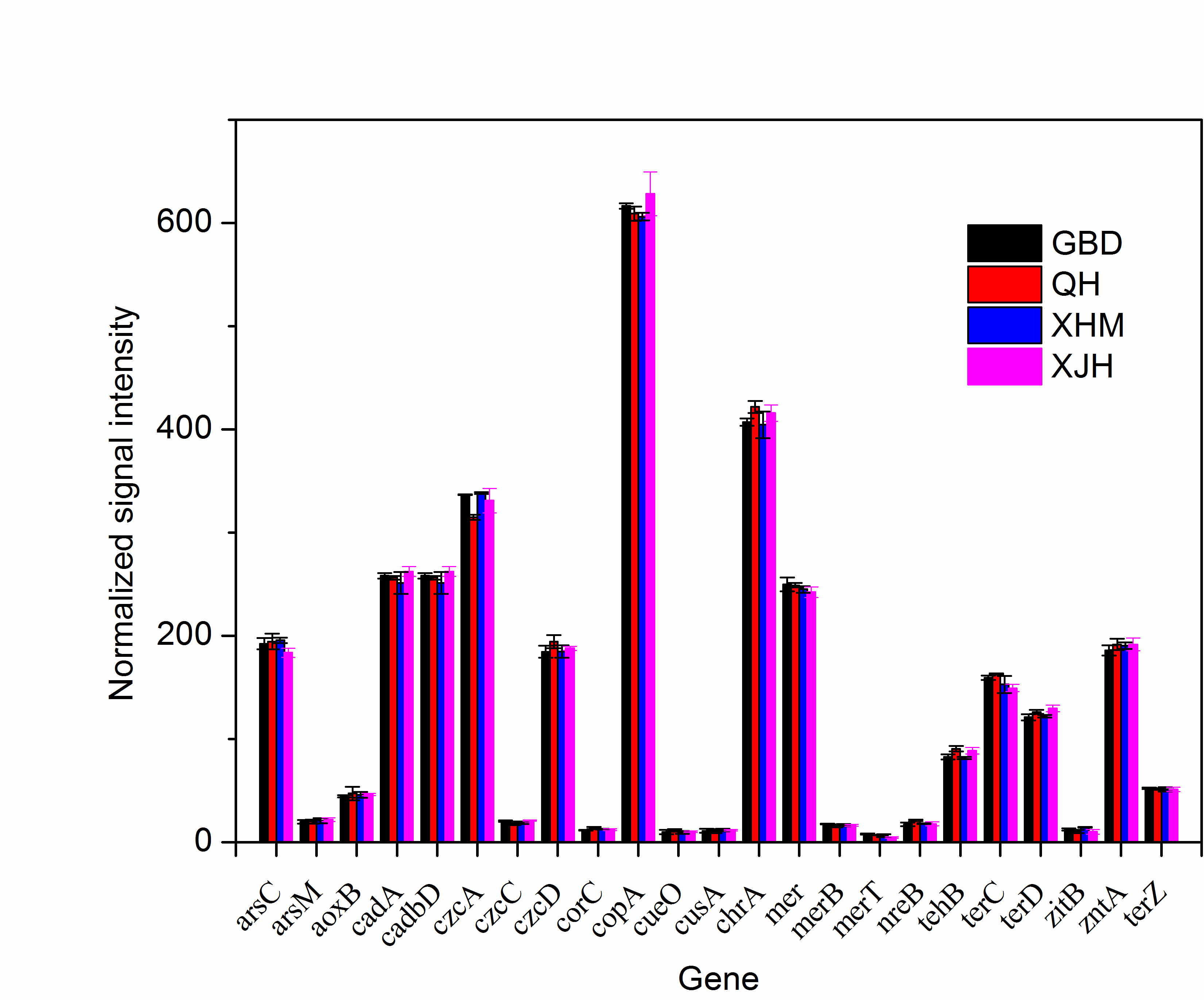


Figure S6 The normalized signal intensity of detected key genes involved in metal resistance. The signal intensity for each functional gene is the average of signal intensities from all the replicates. All data are presented as mean ± SE.

Figure S7 The normalized signal intensity of detected key genes involved in Antibiotic resistance. The signal intensity for each functional gene is the average of signal intensities from all the replicates. All data are presented as mean ± SE.


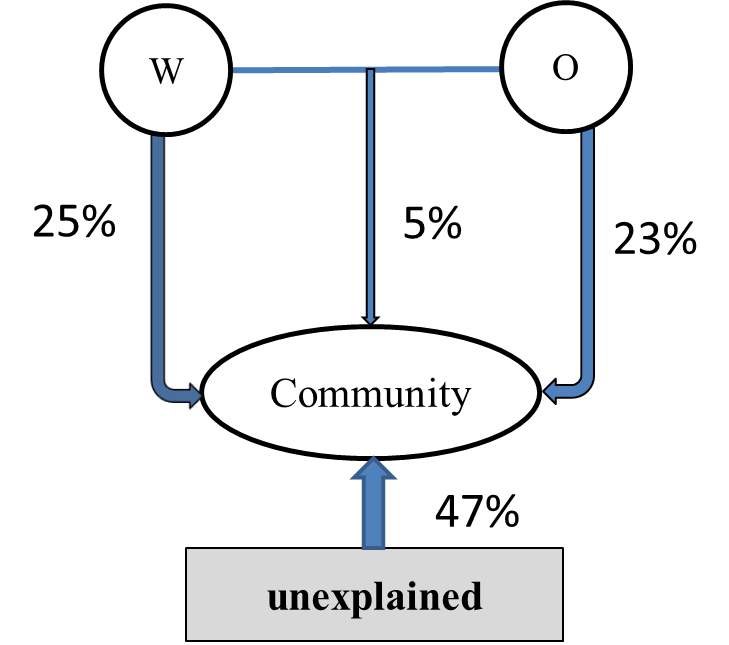


Figure S8 Variation partitioning analysis of microbial diversity explained by wastewater characteristics (W) and operational parameters (O).
